# Internal Pudendal Artery Injury and Bleeding Into the Corpora Cavernosum Due to Complex Open Book Pelvic Fracture

**DOI:** 10.7759/cureus.82277

**Published:** 2025-04-14

**Authors:** Joanna E Jayakumar, Mohammad Zaid, Raya Flayyih, Ahmad Kamal Ansari, Kais Kotiesh, Yaser Saeedi

**Affiliations:** 1 Surgery, Mohammed Bin Rashid University of Medicine and Health Sciences, Dubai, ARE; 2 Urology, Dubai Health, Dubai, ARE; 3 Research, Dubai Medical College for Girls, Dubai, ARE; 4 General Surgery, Rashid Hospital, Dubai, ARE

**Keywords:** bladder trauma, hemodynamic instability (hdi), open pelvic fracture, pelvic hemorrhage, road traffic accident

## Abstract

Open book fractures frequently occur in road traffic accidents and are often associated with organ injuries, soft tissue damage, and urogenital trauma. Pelvic fractures can vary in severity, with some being stable and others involving significant structural disruption. A 32-year-old man presented with severe lower abdominal and right lower limb pain following a road traffic accident. He remained alert and denied loss of consciousness but exhibited tachycardia. Examination revealed lower abdominal tenderness, pelvic swelling, bruising, and a scrotal hematoma. CT imaging showed extensive pelvic fractures involving the sacral promontory and right sacral ala, along with bladder rupture and urethral injury. He underwent urgent pelvic external fixation and exploratory laparotomy with bladder repair. Postoperatively, he developed hypovolemic shock, requiring ICU admission, fluid resuscitation, and blood transfusion. Intensive monitoring and conservative management stabilized his condition, allowing for additional surgical procedures. Despite the severity of his injuries, he showed signs of recovery, with stabilized vital signs and reduced pelvic swelling.

Continued medical and supportive care underscored the importance of timely and comprehensive management in severe pelvic trauma cases. Bladder trauma is a serious complication of pelvic injuries, ranging from minor contusions to complete rupture. These injuries require prompt recognition and surgical intervention. Damage often occurs due to force transmission to the bladder, especially when it is full, leading to increased pressure and potential rupture. Given its location near the pelvic bones, the bladder is at a higher risk of injury in severe trauma. Symptoms may include lower abdominal pain, swelling, bruising, and signs of circulatory distress. Imaging helps assess the extent of injury and guides surgical management. Timely intervention is essential to prevent complications such as infection and long-term dysfunction. A multidisciplinary approach, vigilant monitoring, and aggressive management of complications are key to optimizing recovery in complex pelvic trauma cases.

## Introduction

Open book pelvic fractures typically occur due to high-intensity anteroposterior compression forces, leading to the opening of the pelvis around its craniocaudal axis. This results in increased pelvic volume and potential for significant blood loss in the retroperitoneal space. Unstable pelvic fractures are typically the result of high-energy trauma, such as motor vehicle accidents or falls from significant heights. These fractures involve a complete disruption of the posterior sacroiliac complex, which is crucial for maintaining the stability of the pelvic ring. As a result, these injuries are often associated with severe trauma and can lead to significant complications, including hemorrhage and damage to surrounding organs and tissues. The management of unstable pelvic fractures often requires immediate and aggressive intervention to control bleeding and stabilize the fracture, which may include the use of pelvic orthotic devices or external pelvic fixation [1]. Open book fractures are a specific type of pelvic fracture classified as partially stable (type B). They are characterized by a separation of the pubic symphysis, resembling an "open book," and are typically caused by external rotation forces. While they do involve some instability, the posterior sacroiliac complex remains partially intact, which differentiates them from the more severe unstable fractures. The treatment of open book fractures may involve surgical stabilization, especially if the separation is significant, to restore the normal anatomy and function of the pelvis [2]. Two or more breaks in the pelvic ring are needed to create an unstable pelvic fracture [3]. Most pelvic hemorrhages are thought to be caused by injury to small arteries or veins in the fractured cancellous pelvic bone or the surrounding soft tissues, and only 6%-18% of patients with unstable pelvic fractures have hemorrhage from larger arteries [4]. Although arterial bleeding accounts for only 10% of total pelvic hemorrhage, it is more frequently associated with hemodynamic instability than other causes [5]. An open pelvic fracture is a devastating injury. A graded approach to managing hemorrhage that includes wound packing, anti-shock trousers, angiographic embolization, and hemipelvectomy should be used [6]. The mortality rate reported can be as high as 50% in some cases [4].

## Case presentation

A 32-year-old man, previously healthy, presented with severe lower abdominal and right lower limb pain immediately after a road traffic accident. Despite the trauma, he remained alert and denied any loss of consciousness, although he exhibited tachycardia. Upon examination, lower abdominal tenderness was noted, along with significant pelvic swelling accompanied by bruising and a hematoma of the scrotum.

**Figure 1:**
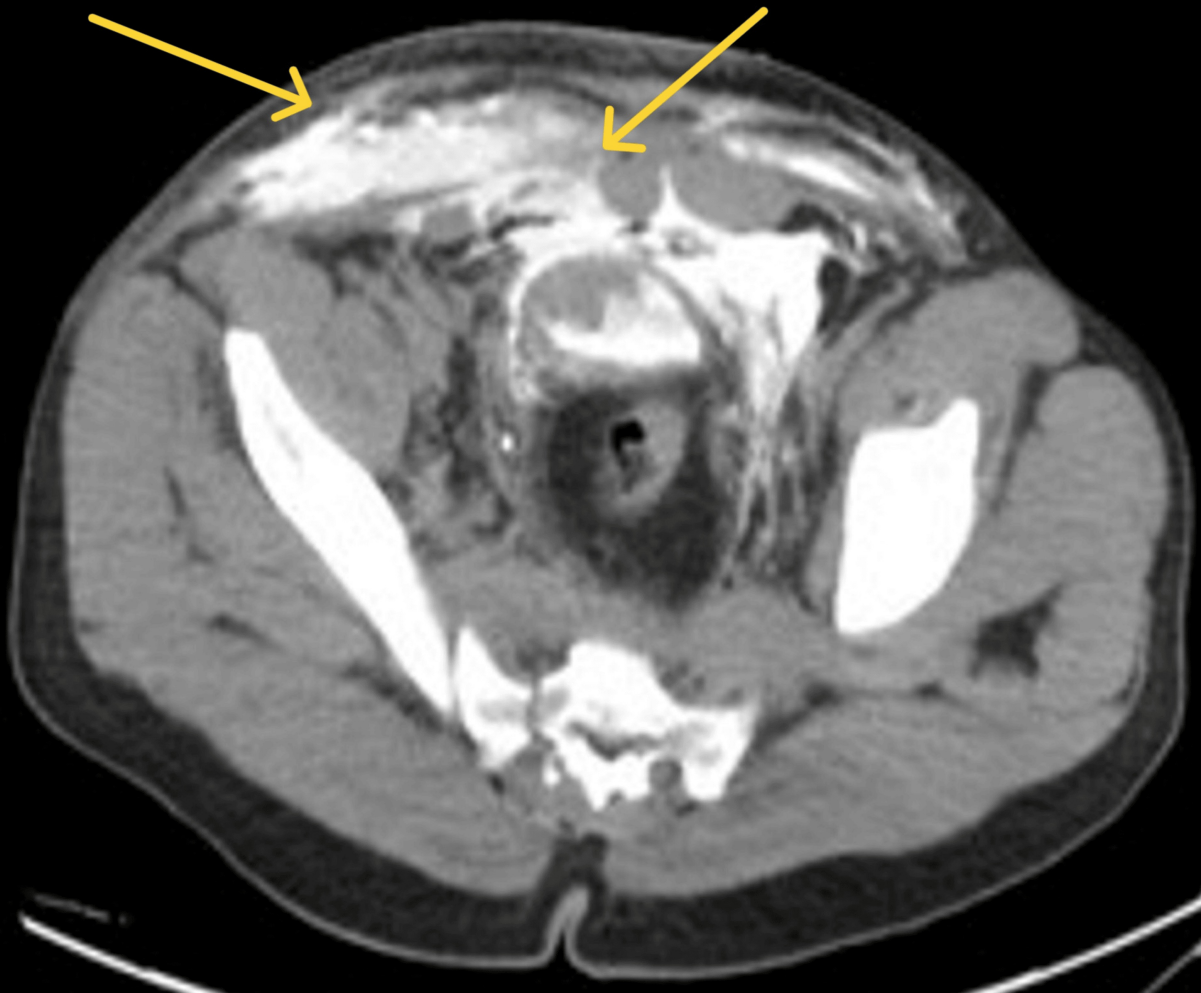
Anterior and anterior inferior defect in the urinary bladder wall with extraperitoneal contrast extravasation, leaking along the anterior abdominal wall, multiple blood clots in the urinary bladder, and a small amount of contrast in the left paracolic gutter Impression: Combined intraperitoneal and extraperitoneal bladder rupture and associated urethral injury

**Figure 2:**
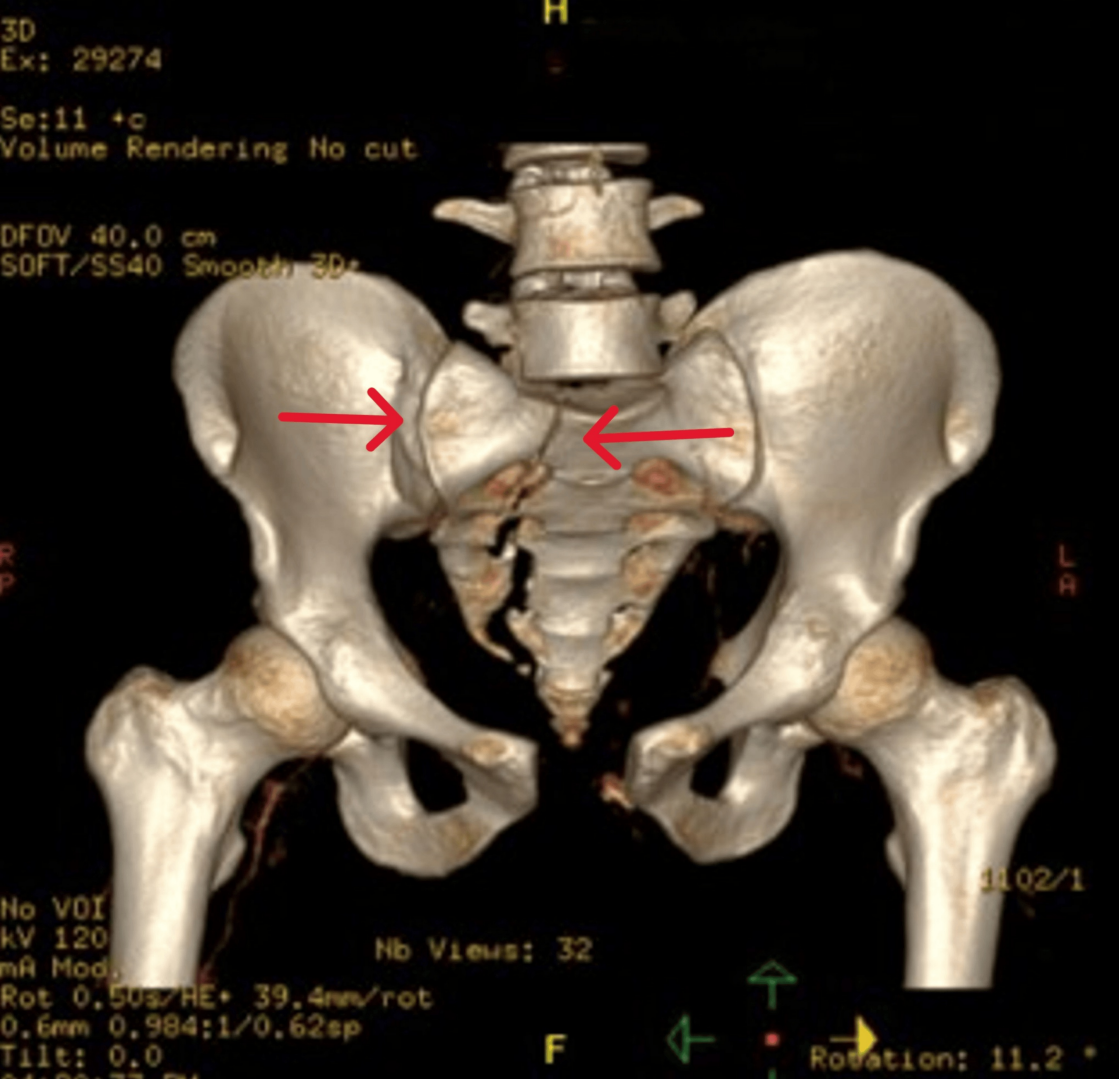
Fractures of the sacral promontory and right sacral ala and widening of the right sacroiliac joint and pubic symphysis Impression: Combined intraperitoneal and extraperitoneal bladder rupture and associated urethral injury

Further investigations, including CT scans, revealed extensive pelvic fractures, notably involving the sacral promontory and right sacral ala, along with bladder rupture and urethral injury (Figures 1, 2). The patient underwent urgent surgery, including pelvic external fixation and exploratory laparotomy with bladder repair. However, postoperatively, he developed hypovolemic shock due to inappropriate pelvic fixation, necessitating re-exploration by the trauma team. Then, he was taken for admission to the ICU for aggressive fluid resuscitation and blood transfusion.

In the ICU, the patient was closely monitored, and conservative management strategies were employed to stabilize his condition. After stabilization, he underwent additional surgical procedures. Despite the severity of his injuries, the patient showed promising signs of recovery in the postoperative period. His vital signs stabilized, and the swelling in the pelvic region gradually subsided. With continued medical attention and supportive care, the patient's condition improved, highlighting the importance of prompt and comprehensive management in cases of severe pelvic trauma.

## Discussion

Bladder trauma and rupture are severe complications that can arise in the context of significant pelvic injuries, as exemplified by the case of a 32-year-old male victim of a road traffic accident. These bladder injuries can range from minor contusions to complete rupture, often necessitating prompt diagnosis and surgical intervention [4].

The mechanism of bladder trauma in the setting of pelvic fractures involves the transmission of the forceful impact to the bladder, particularly when the organ is distended, leading to an increase in intravesical pressure that exceeds the bladder's tensile strength [4]. Pelvic fractures are disruptions of the bony structures of the pelvis, including pelvic ring fractures, acetabular fractures, and avulsion fractures [7]. The proximity of the bladder to the fractured bony structures, such as the sacral promontory and right sacral ala in this case, further heightens the risk of bladder injury.

Clinical presentation may include severe lower abdominal pain, pelvic swelling, bruising, and scrotal hematoma, accompanied by tachycardia as a potential indicator of hypovolemia due to blood loss [4]. Diagnostic imaging, primarily CT scans, plays a crucial role in confirming the diagnosis and delineating the extent of the injury, including associated pelvic fractures and concomitant urethral involvement [3].

Extraperitoneal rupture accounts for 60% of all bladder injuries and is typically treated conservatively with a Foley catheter [8]. Intraperitoneal ruptures, which make up about 30% of bladder ruptures, require surgical treatment [8].

Prompt surgical management, involving procedures such as exploratory laparotomy and bladder repair, is essential to prevent life-threatening complications such as urinary peritonitis and sepsis, as well as to mitigate long-term bladder dysfunction [4]. However, the risk of postoperative complications, such as hypovolemic shock, underscores the need for comprehensive, multidisciplinary care to optimize patient outcomes in these complex pelvic trauma cases [3].

Age-related differences in pelvic fracture outcomes are a critical consideration when assessing and treating patients. Studies have shown that older patients, typically those over 65, tend to have higher mortality rates and different risk factors compared to younger patients with similar injuries [3,5]. This underscores the need for age-specific management strategies and risk assessment tools in treating pelvic fractures. For instance, elderly patients with pelvic fractures have been found to have longer hospital stays and a higher likelihood of dying from multisystem organ failure compared to younger patients [5].

Regional variations in pelvic trauma management highlight the importance of considering different healthcare systems and populations when developing treatment protocols. Comparing findings from various regions can reveal potential differences in incidence rates, management approaches, and outcomes [4,6]. For example, a study of international orthopedic trauma surgeons revealed variations in the use of emergency stabilization techniques and hemostatic procedures across different regions [4]. These comparisons can inform best practices and areas for improvement in pelvic trauma care globally, potentially leading to more standardized and effective treatment algorithms.

## Conclusions

Pelvic fractures with bladder trauma require early recognition, rapid stabilization, and a multidisciplinary approach to prevent severe complications. Timely surgical intervention, along with intensive postoperative care, is crucial for recovery. Close monitoring for shock, infection, and delayed healing enhances patient outcomes. Factors such as age, injury severity, and access to specialized care influence recovery, highlighting the need for individualized management. Advances in trauma care continue to refine treatment approaches, emphasizing early intervention and comprehensive rehabilitation. A well-coordinated strategy, incorporating surgical expertise and long-term rehabilitation, is essential to improving survival rates, minimizing complications, and optimizing functional recovery in pelvic trauma patients.
